# Supplementary material for: Pre-initiation and elongation structures of full-length La Crosse virus polymerase reveal functionally important conformational changes
Source: Nat Commun. 2020 Jul 17;11:3590. doi: 10.1038/s41467-020-17349-4 (PMC7368059; doi:10.1038/s41467-020-17349-4)
Supplement: Supplementary file 1 — Supplementary Information [file 41467_2020_17349_MOESM1_ESM.pdf]

SUPPLEMENTARY INFORMATION

Pre-initiation and elongation structures of full-length La Crosse virus polymerase reveal functionally important conformational changes

Benoît Arragain et al.

Supplementary Table 1 Data collection and refinement statistics

	**LACV L FL Pre-initiation** (6Z6B)
**Data collection**	
Beamline (ESRF)	ID29
Wavelength (Å)	0.9724
Space group	C2
Cell dimensions	
a, *b*, *c* (Å)	371.19, 145.32, 234.19
α, β, γ (°)	90, 116.33, 90
Resolution (Å)	172.95-3.96 (4.35-3.96) *
R_{meas}	0.163 (1.285)
$I / \sigma I$	6.5 (1.6)
CC(1/2)	0.995 (0.470)
Completeness (%)	
Spherical	60.8 (12.4)
Ellipsoidal	91.3 (55.8)
Redundancy	11.6 (13.3)
**Refinement**	
No. reflections work/free	57697/1514
R_{work}	0.264 (0.323)
R_{free}	0.299 (0.370)
No. protein atoms	34640
Average B-factors (Å^2)	189.8
R.m.s. deviations	
Bond lengths (Å)	0.002
Bond angles (°)	1.156
**Validation**	
Ramachandran favoured	90.0
Ramachandran outliers	1.04
Molprobity score	2.11
Clash score	3.07

*Values in parentheses are for highest-resolution shell.

Supplementary Table 2: Cryo-EM data collection, refinement and validation statistics

	**LACV L<br>Pre-initiation**<br><br>(6Z6G)<br>(EMD-11093)	**LACV L<br>CBD**<br><br>(6Z6G)<br>(EMD-11095)	**LACV L<br>ZBD without<br>β-hairpin**<br>(6Z6G)<br>(EMD-11107)	**LACV L<br>Elongation**<br><br>(6Z8K)<br>(EMD-11118)
**Data collection and processing**				
Microscope	ThermofischerTitan Krios			
Camera	Gatan K2 Summit + GIF			
Magnification	165000			
Voltage (kV)	300			
Number of frames	40			
Electron exposure (e^-/A^2)	50			
Defocus range (μm)	0.8-3.5			
Pixel size (Å)	0.4135 in super-resolution, 0.827 used for processing			
Symmetry imposed	C1			
Initial/Final micrographs (no.)	16498/16015			
Final particles (no.)	57660	131058	51842	59152
Map resolution (Å) 0.143 FSC threshold	3.06	3.54	3.49	3.02
Map resolution range (Å)	2.9-5	3.5-4.3	3.4-4.6	2.9-5
**Refinement**				
Map content during refinement	Entire pre-initiation map	CBD and mid domains	ZBD and mid domains	Entire elongation-mimicking map
Protein/ligand content during model refinement<br>For RNA, if present but not built due to flexibility: indicated in italics	-Protein: endonuclease, core, mid thumb ring linker, mid, ZBD β-hairpin (residues 1-1841, 1978-2025, 2084-2102)<br>-RNA:<br>*5′ vRNA (1-10): 5′-AGUAGUGUGC-3′<br>*5’ vRNA (9-16): 5′ GCUAC*CAA*-3′<br>*3’ vRNA (1-16): 3′-UCAUCACAUGA*UGGUU*-5′	-Protein: residues 1842-1977	-Protein: residues 2026-2083, 2103-2263<br><br>-Zinc ion	-Protein: same residues as in Pre-initiation<br>-RNA:<br>*5′ vRNA (1-10): 5′-AGUAGUGUGC-3′<br>*5’vRNA (9-16): 5′ GCUAC*CAA*-3′<br>*3’vRNA (1-16): 3′-UCAUCACAUGA*UGGUU*-5′<br>*Template mimicking RNA: 5′-AGUAGUGUGC-3′<br>*Product mimicking RNA: 3′-UCAUCACAUG*AUGGUU*-5′
Initial model used	5AMQ	-	-	5AMQ
Model resolution (Å) 0.5 FSC	3.06	4.03	3.80	3.12

threshold				
Map sharpening B factor (Å^2)	-40	-90	-83	-40
Model composition				
Protein residues	1815	129	201	1831
Ligands	29	0	1	46
B-factor (Å^2)				
Protein min-max (mean)	12.93-145.07 (55.74)	69.72-127.37 (93.35)	55.55-135.31(84.74)	40.59-158.48 (74.67)
R.m.s deviations				
Bond lengths (Å)	0.008	0.005	0.005	0.009
Bond angles (°)	0.889	0.924	0.844	0.931
Validation				
MolProbity score	1.46	1.88	1.80	1.54
Clashscore	3.57	4.97	3.99	3.79
Poor rotamers (%)	0.24	0.00	0.53	0.53
Ramachandran plot				
Favored (%)	95.53	87.07	93.85	94.57
Allowed (%)	4.47	12.97	6.15	5.43
Disallowed (%)	0	0	0	0

Supplementary Table 3 codon-optimized gene sequence
atgggccaccatcatcaccaccatgattatgatattccaactaccgagaatttgtattttcaggggatggactaccaggaatatcaacagtttctggcacgtatc
aacaccgcacgtgacgcttgcgtggccaaggatattgatgtcgacctgctgatggcccgtcacgactatttcggacgcgaactctgtaaaagcttgaacatc
gaataccgtaacgacgtgcctttcatcgacatcattctggacattcgtcctgaggttgacccgctgaccatcgacgcaccgcacatcacccctgataactatct
gtacattaataacgtgctgtacatcatcgattacaaggttagcgtctccaatgaaagcagcgtcattacgtacgacaagtactacgaactgacccgcgatatct
ccgaccgtctgagcatcccgatcgaaattgttattatccgtattgatccagtgagccgcgatctgcatatcaactccgatcgtttcaaggaattgtatccgactat
cgtggtcgatattaactttaaccagttctttgacctcaagcaactgctgtacgaaaagttcggcgatgacgaggaatttctgctgaaggtcgcacatggtgatttt
accttgactgccccgtggtgtaagacgggctgtccggaattttggaagcacccgatttacaaggagttcaagatgagcatgccggtgcccgaacgtcgtctg
ttcgaggagagcgtcaaattcaacgcgtacgagtccgagcgctggaacaccaacctggttaaaatccgtgaatacactaaaaaggactatagcgagcacat
tagcaagtctgcgaaaaacattttcctggcgtccggtttctacaagcaacctaacaaaaacgagatttctgaaggctggaccctgatggttgagcgtgtccag
gaccaacgcgagatctctaaaagcctgcacgatcaaaagccctctatccatttcatctggggtgcccacaacccgggaaacagcaacaatgccacgttcaa
gttgatcctgctgagcaaaagcttgcagagcattaaaggaatctccacctacaccgaggcttttaaatccctgggcaaaatgatggatattggtgacaaggca
atcgagtacgaggagttctgcatgagcctgaaaagcaaggcgcgcagcagctggaagcagattatgaataagaagctggaacctaagcagatcaacaac
gcactggtcctgtgggaacaacagtttatgatcaacaatgacctgatcgataaaagcgaaaagctgaagctcttcaaaaatttctgcggcattggtaaacaca
agcagttcaagaacaagatgctggaggacctggaggtcagcaaaccgaagattctggattttgacgacgccaatatgtacctggccagcctgaccatgatg
gaacagagcaaaaagatcctgtccaagagcaatggtctgaaacctgacaactttatcttgaacgaattcggcagccgtattaaagatgcaaacaaggaaac
gtacgataatatgcacaaaatctttgaaacgggttattggcaatgtatcagcgatttcagcacgctcatgaagaacatcttgagcgtcagccaatataatcgcc
acaatacctttcgcattgccatgtgcgcgaacaataacgtcttcgcgatcgtcttcccgagcgccgacattaaaaccaaaaaggctacggtggtttacagcat
cattgttctgcacaaggaggaagaaaacatcttcaatcccggctgtctgcacggtaccttcaagtgcatgaatggctacatcagcatctctcgcgccatccgc
ctggataaggagcgttgccaacgcatcgtgagctccccaggactctttctgaccacctgcctgctcttcaagcatgataaccccacgctggtgatgagcgat
attatgaactttagcatttacaccagcctgtccattaccaagtctgtcttgagcttgaccgagccagcccgttatatgatcatgaactctctggctatcagcagca
acgtcaaggactatatcgcggaaaaattctctccgtacacgaagaccctgttctccgtctacatgacccgcttgattaagaatgcgtgcttcgacgcatacgat
cagcgtcagcgcgttcaactgcgcgacatttacttgtctgactatgatatcacccaaaaaggtatcaaagacaatcgcgaattgacctccatctggttccccgg
cagcgttacgttgaaggaatatctgacccagatctacctgccattttacttcaatgccaagggcctgcatgagaagcatcacgtcatggtggacctggcgaag
actatcctggagatcgagtgtgagcagcgtgaaaatatcaaggagatctggagcactaattgcacgaaacagaccgttaacctcaagatcctgattcactcc
ctgtgcaaaaatctgctggcggacacgtctcgtcataaccatctgcgcaaccgcattgaaaaccgtaataacttccgtcgttctatcacgaccatcagcacttttt
acgtccagcaagtcttgtctgaagattggtgactttcgtaaagaaaaggagctgcaatccgttaagcagaagaagatcctggaggtgcaatcccgtaagatg
cgtttggctaatccgatgtttgtgacggacgagcaggtttgcctggaggttggccattgcaactacgaaatgctccgtaacgcaatgccgaattacaccgatt
acatttccaccaaggtctttgatcgcctgtacgaactgttggacaagaaagtcctgacggataaacctgtgattgagcagatcatggatatgatgatcgaccac
aagaagttctatttcactttctttaacaaaggccagaaaacgtctaaagaccgcgaaattttcgtcggtgaatatgaagctaaaatgtgcatgtacgcggtgga
acgtatcgctaaagagcgttgcaaactcaacccagacgaaatgattagcgagccgggagacggtaagctgaaagtgctggaacaaaagtccgagcaaga
aatccgtttcctcgttgagaccactcgccagaaaaaccgtgaaatcgacgaagccatcgaggctttggctacggaaggatacgaaagcaacctgggtaaa
atcgagaaactgtctctgggtaaggcgaaaggcctgaagatggagatcaacgccgatatgagcaaatggagcgctcaggatgtgttctataaatacttctgg
ttgatcgcgctggacccgattttgtatccgcaggagaaggaacgcattctctacttcatgtgcaattacatggacaaggagctgatcctgccagacgagctgc
tgtttaatctgttggaccaaaaggtggcgtaccagaatgacattatcgcgaccatgaccaatcaactgaattccaacaccgtgctgattaaacgtaactggctg
cagggtaatttcaactacacttctagctacgttcacagctgcgcgatgagcgtctacaaagagatcttgaaagaggctattactctcctggacggctctatcttg
gttaacagcctcgttcattctgatgataaccagacctctatcaccattgtccaagataaaatggagaacgacaagatcatcgacttcgccatgaaagagttcga
acgtgcttgcctgacgtttggctgtcaggcgaatatgaagaaaacctacgtcaccaattgcatcaaggagtttgttagcctgtttaacctgtatggtgagccgtt
cagcatttacggccgcttcttgctgaccagcgtcggcgactgcgcgtacattggtccttatgaggatctggcaagccgcatcagctccgcccagaccgcaat
caagcacggttgcccgccgagcctggcttgggttagcatcgccattagccactggatgacgagcctgacttacaatatgctgccgggtcagagcaatgacc
cgatcgattactttccggcggagaaccgtaaggacatccctatcgaactgaacggtgttctggacgctcctttgtctatgatttccaccgtgggtctggaaagc
ggaaatctgtatttcctgatcaagctcctgagcaaatatacgccggtgatgcaaaagcgtgagtccgttgttaaccagattgcagaggtcaagaattggaagg
ttgaggacttgactgacaacgaaatctttcgcctcaaaatcctccgctacttggttctggatgcggagatggacccgagcgacatcatgggcgaaacctccg
acatgcgcggtcgtagcattctgacgccacgtaaattcacgaccgcgggtagcctccgtaagttgtacagctttagcaagtaccaggatcgtttgtcctcccc
gggtggtatggtggagctgttcacgtatctgctggagaaacctgagctgctggttactaagggcgaggacatgaaagactacatggaaagcgtgattttccg
ctacaacagcaaacgttttaaagaatccttgagcattcagaaccccgcgcaactgttcattgagcaaattctgttcagccacaagccggtcattgacttttccgg
cattcgcgataaatacattaatttgcatgactcccgcgctctggaaaaggaaccggacatcctcggtaaagttacgttcacggaagcgtaccgtctgctgatg
cgtgatttgagctctctggagctgactaacgatgacatccaggtcatctactcctatattatcctgaacgacccgatgatgatcaccatcgcgaacacccacat
cttgtccatttacggttccccgcaacgccgtatgggcatgtcctgcagcacgatgccggaatttcgtaatctgaagctgattcaccacagcccagccctggtc
ctgcgtgcctattctaagaacaatccggatatccaaggtgccgatccgaccgaaatggcccgtgacctggtgcatctgaaggaattcgtcgaaaacactaac
ctggaagaaaagatgaaagtccgcatcgctatgaatgaagctgagaagggtcaacgtgacattgttttcgagctgaaggagatgacgcgcttttaccaagttt
gctacgagtacgtgaaatctacggagcacaagatcaaagtcttcatcctccccgcgaagagctataccaccacggacttctgctccctgatgcagggcaatt
tgattaaagacaaagaatggtacaccgtccactacttgaaacagattctgtccggtggccataaggctatcatgcaacacaacgcgaccagcgagcagaac

atcgcttttgagtgcttcaagctgatcactcactttgcggatagcttcattgacagcttgtctcgtagcgcgttcctgcagctgatcattgacgagtttagctataa
agacgttaaagtgagcaagttgtacgacattattaagaacggctacaaccgtaccgatttcatccccctgctgttccgtactggtgacctgcgccaggcagac
ctggataaatacgacgcaatgaagtcccacgagcgcgtcacctggaacgattggcaaacgtcccgtcatttggacatgggctccattaacctgaccattacc
ggctataaccgtagcatcactatcatcggtgaggacaataagttgacctacgccgagctgtgtctgacgcgcaagactcctgaaaacattaccatctccggtc
gtaaattgctgggcagccgtcatggtttgaagttcgaaaacatgagcaagatccagacctatccgggtaactactatattacctatcgtaaaaaggatcgtcat
cagttcgtctaccaaatccactcccacgaaagcattacgcgtcgtaacgaagagcacatggcgattcgtacccgtatctacaatgagattacgccggtctgtg
ttgttaatgtcgccgaggtggacggagatcagcgtattctgattcgtagcctggactatttgaataatgacatttttagcctctcccgcattaaagttggtctggac
gaattcgctaccatcaagaaagcgcattttagcaagatggtgtctttcgagggacctcccattaagactggtctgctggatctgaccgagctgatgaagagcc
aggatctgttgaatctgaattacgacaacattcgcaatagcaacctgatttccttctctaaactgatctgctgcgagggtagcgataacatcaacgacggtctg
gagttcctgagcgacgatccgatgaatttcaccgagggcgaggcaatccacagcaccccgattttcaatatttactattctaaacgtggtgaacgtcacatga
cctaccgtaatgcgattaaactgctgatcgagcgtgaaaccaagatctttgaggaggccttcacgtttagcgaaaatggcttcattagccctgaaaacctggg
atgcctggaagccgtcgtgagcctcatcaagctgctgaagacgaatgagtggtctaccgtgatcgacaaatgtattcacatctgtctcatcaagaacggaatg
gatcacatgtaccacagcttcgacgtgccgaaatgttttatgggaaacccgattactcgtgacattaactgggtcatgttccgtgagtttatcaacagcctgccg
ggtaccgacatcccgccgtggaacgtcatgactgagaacttcaagaagaaatgtatcgctctgattaacagcaaatttgagacgcaacgcgacttctctgag
tttacgaaactgatgaaaaaggagggcggtcgctccaacattgagttcgactaatga

Supplementary Figure 1

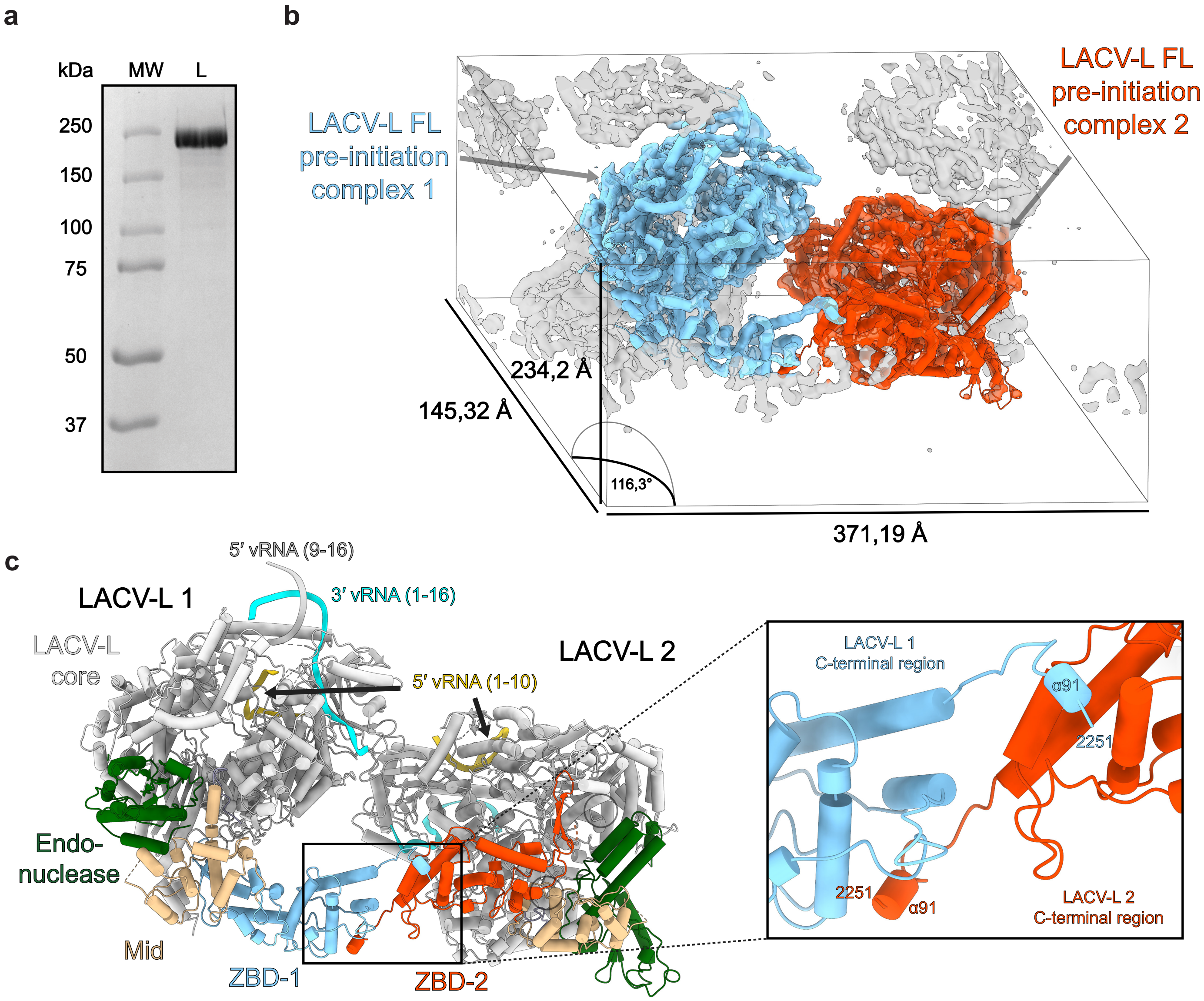

Supplementary figure 1: Purification and X-ray crystal structure

a, 8% SDS-PAGE gel of LACV-L FL purification after gel filtration step (MW: Molecular weight; L: LACV-L FL). **b**, Electron density map of one asymmetric unit. The two polymerase complexes at pre-initiation are respectively colored in blue, red and labelled. Asymmetric unit dimension and angles are displayed. **c**, X-ray crystallography structure shown in the same orientation as in b displaying the 2 polymerase complexes of the asymmetric unit. The core regions are colored in white, the endonucleases in green, the mid domains in beige and the ZBD according to LACV-L 1/LACV-L 2 colors in blue and orange. The swap of the last α-helix 91 is shown in a close up view and the C-terminal residue 2251 is labelled. The RNA promoter positions are shown.

Supplementary Figure 2

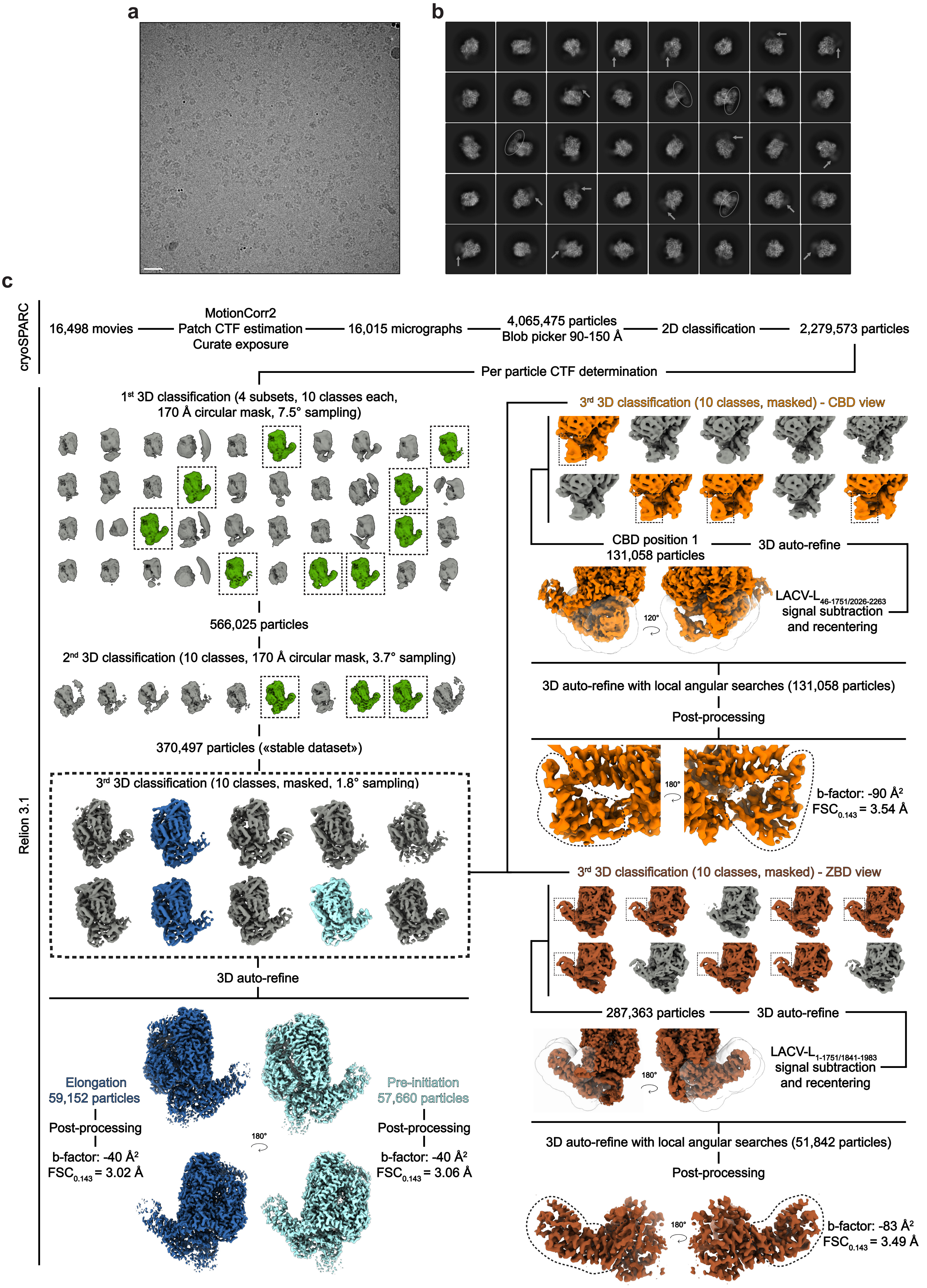

Supplementary figure 2: Cryo-EM data collection and image processing

a, Cryo-electron micrograph of LACV-L FL collected on the ESRF CM01 Titan Krios equipped with a K2 direct electrons detector camera at -2 µm defocus. Scale bar = 200 Å. **b**, Representative 2D classes of LACV-L FL. Flexibility of the LACV-L C-terminal region seen in some class averages is highlighted with white arrows. Stabilized LACV-L C-terminal regions visible in other class averages are surrounded by a white circle. **c**, Cryo-EM image processing pipeline is described in detail in the “Image processing” method section.

Supplementary Figure 3

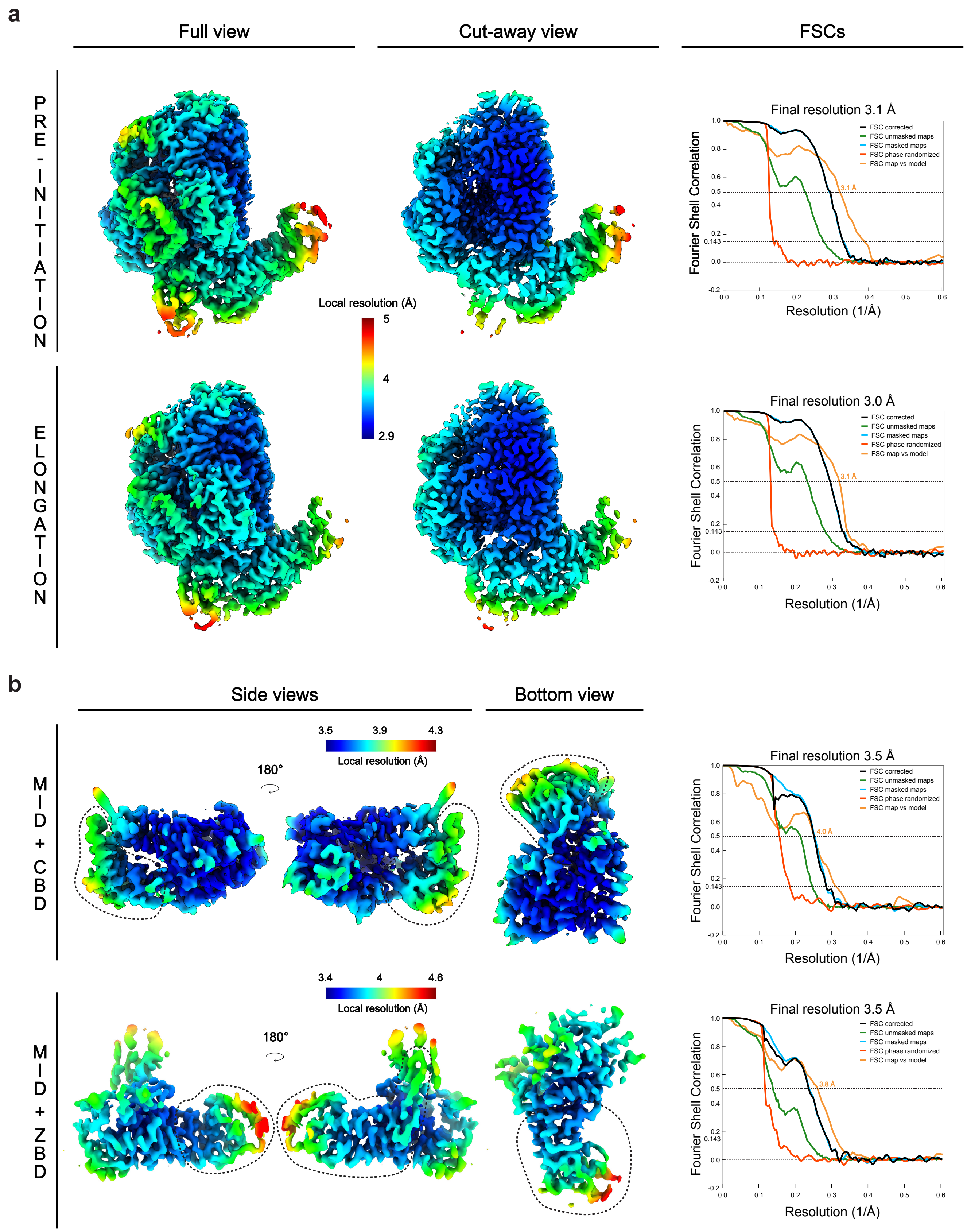

Supplementary figure 3: Cryo-EM maps local resolution and FSCs

a, Full view and cut-away view of the pre-initiation and elongation-mimicking cryo-EM maps. Maps are filtered and colored according to their local resolution. Resolution range coloring is indicated. FSCs corrected, FSCs of unmasked maps, FSCs of masked map and phase randomized FSCs are respectively displayed in black, green, light blue and red. The FSC of the map and the model are displayed in orange. The model refined in the pre-initiation and the elongation-mimicking cryo-EM maps corresponds to the core, the mid domain, the endonuclease domain and the ZBD β-hairpin. The gold-standard Fourier shell correlation (FSC) of masked maps indicates a respective resolution of 3.1 Å and 3.0 Å for the pre-initiation and the elongation-mimicking cryo-EM maps with the FSC = 0.143 criteria. **b**, Side view and bottom view of the subtracted maps containing (i) the CBD and the mid domain, (ii) the ZBD and the mid domain. The maps are filtered and colored according to their local resolution. Resolution range coloring is indicated. The CBD position is surrounded by a dotted line in the map containing the CBD and the mid domain. The ZBD position is surrounded by a dotted line in the map containing the ZBD and the mid domain. FSCs are displayed and colored as in a. Gold-standard Fourier shell correlation of masked maps indicate a resolution of 3.5 Å for both the CBD-mid domain map and the ZBD-mid domain map (FSC = 0.143 criteria).

Supplementary Figure 4

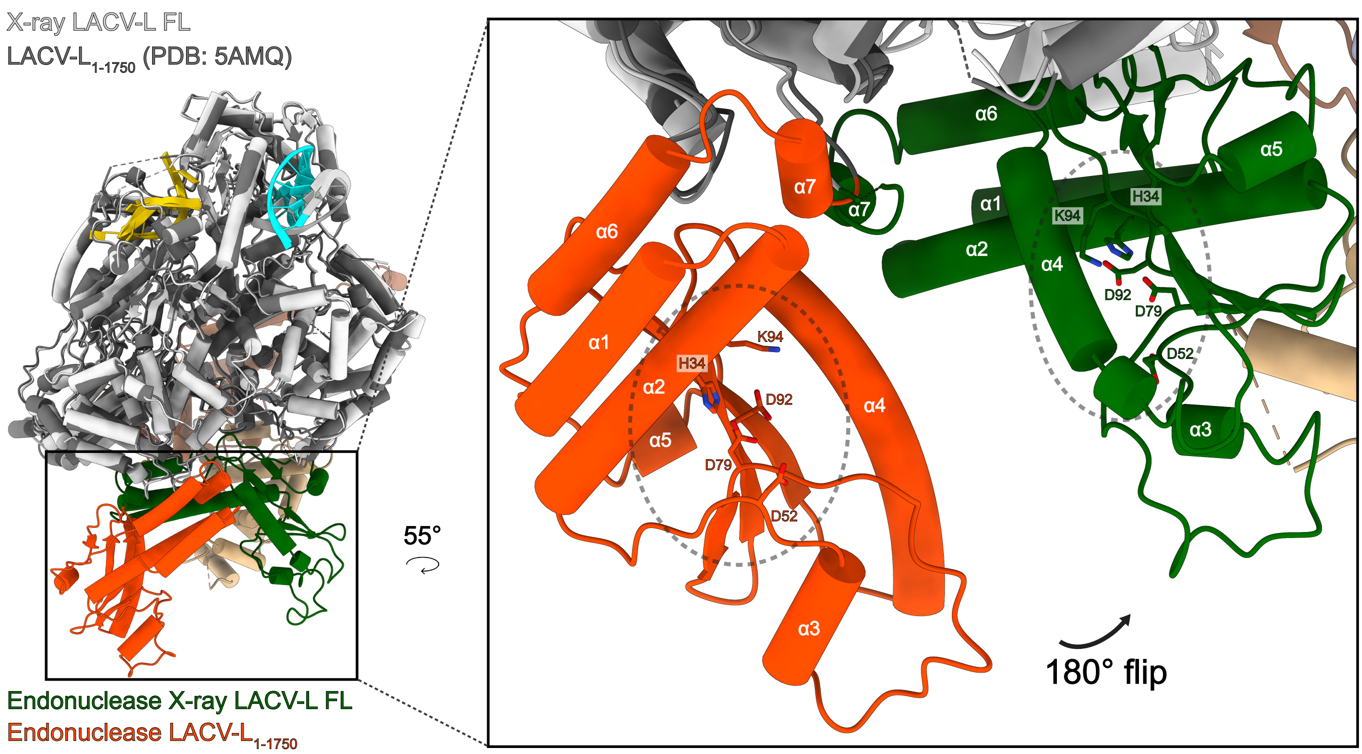

Supplementary figure 4: Endonuclease movement between LACV-L FL and LACV-L$_{1\text{-}1750}$

Superimposition of LACV-L FL and LACV-L$_{1\text{-}1750}$ (PDB: 5AMQ). LACV-L FL core is shown in light grey, LACV-L$_{1\text{-}1750}$ core in dark grey. LACV-L FL endonuclease is shown in green, LACV-L$_{1\text{-}1750}$ endonuclease in red. The 180° rotation of the endonuclease domain is indicated. On the close-up view, the endonuclease active site is surrounded by a dotted ellipse, residues are displayed and α-helices are numbered.

Supplementary Figure 5

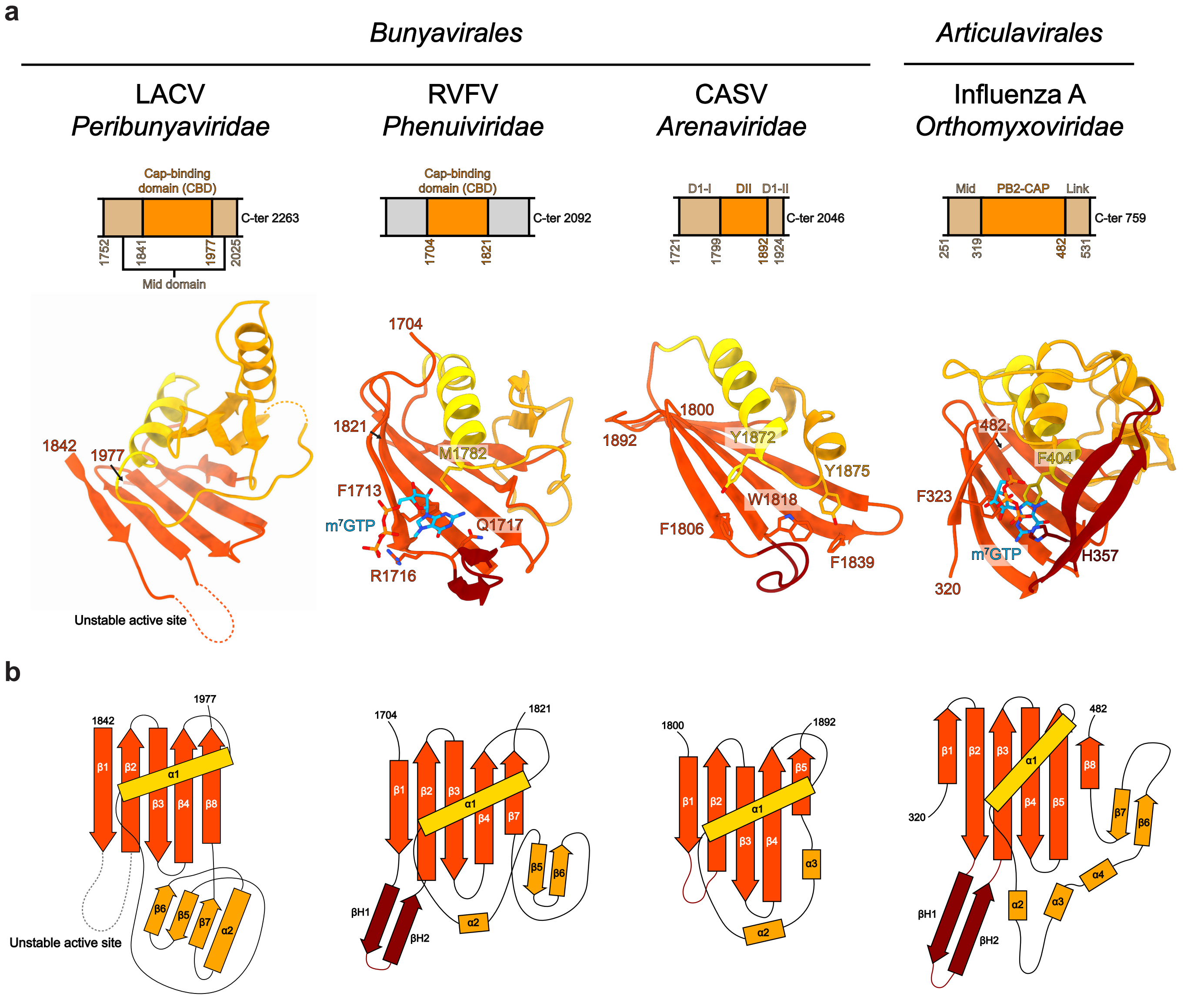

Supplementary figure 5: Structural comparison of sNSV CBD

a, Schematic representation of domains surrounding the CBD of LACV, RVFV, CASV and influenza A virus. Structures of LACV-L CBD, RVFV-L CBD (PDB: 6QHG), CASV-L putative CBD (PDB: 5MUZ) and influenza A PB2-CAP (PDB: 2VQZ) shown as ribbon. Similar structure elements are depicted in the same color: structurally conserved β-sheet in dark orange, α-helix in gold and specific insertions in orange. Residues involved in cap binding are shown and labelled. m^7GTP molecules are colored in cyan. **b**, Schematic representation of CBDs shown as in **a**. LACV-L CBD loop suggested to contain the m^7GTP binding site is shown as a dotted line. For clarity, secondary structures are numbered in the same way starting from α1 and β1.

Supplementary Figure 6

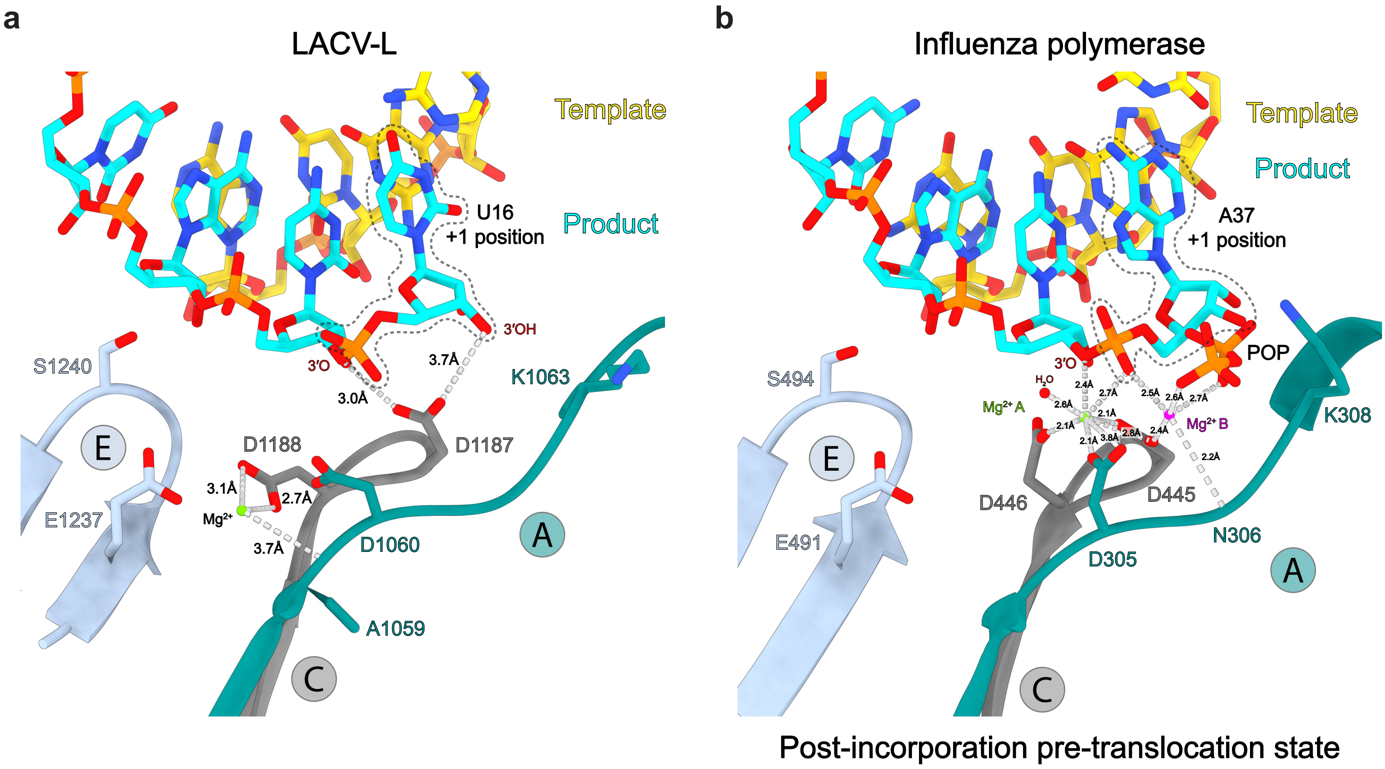

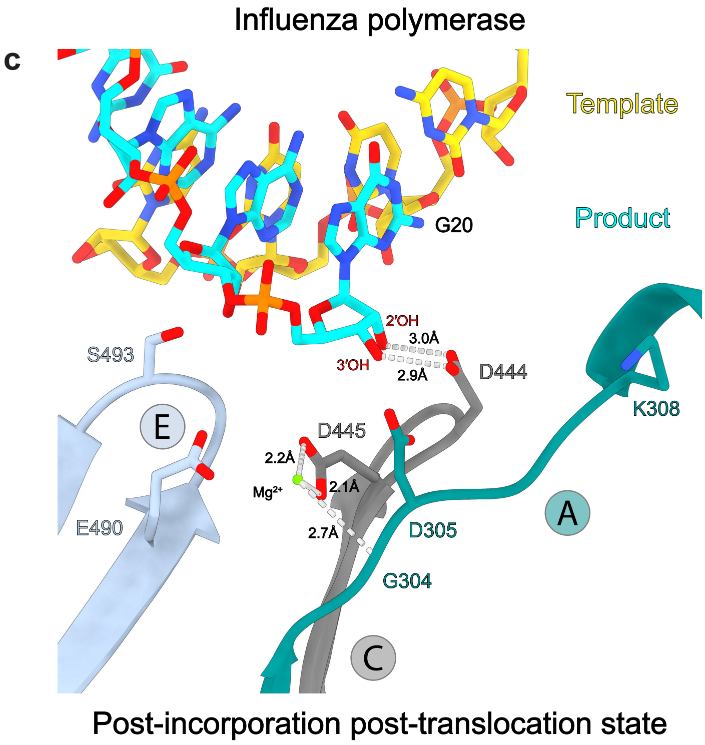

Supplementary figure 6: Comparison between LACV-L and influenza polymerases active sites states.
a, **b**, **c**, The template, product, motif A, C and E are respectively displayed in yellow, cyan, sea green, grey and dark grey. The last incorporated nucleotides are circled by a dotted line. Distances between active site residues and coordinated ion(s) are labelled and shown as grey dotted lines. **a**, LACV-L active site at elongation-mimicking stage. Residues A1059, D1060, D1188 and E1237 coordinate a magnesium ion. D1187 interacts with the 3′O of the previous nucleotide. **b**, Influenza polymerase active site in a post-incorporation pre-translocation state (PDB: 6SZU). Magnesium ion in position A is colored in green, coordinated by D305, D445, D446, 3′O of the previous nucleotide and a water molecule (H_2O) colored in red. Magnesium ion in position B is colored in purple, coordinated by N306, D445, the +1 nucleotide phosphate and a pyrophosphate (POP). **c**, Influenza polymerase active site in a post-incorporation post-translocation state (PDB: 6QCT). Residues G304, D305, D445 and E490 coordinate a magnesium ion. D444 interacts with the 2′OH and 3′OH of the last nucleotide.

Supplementary Figure 7

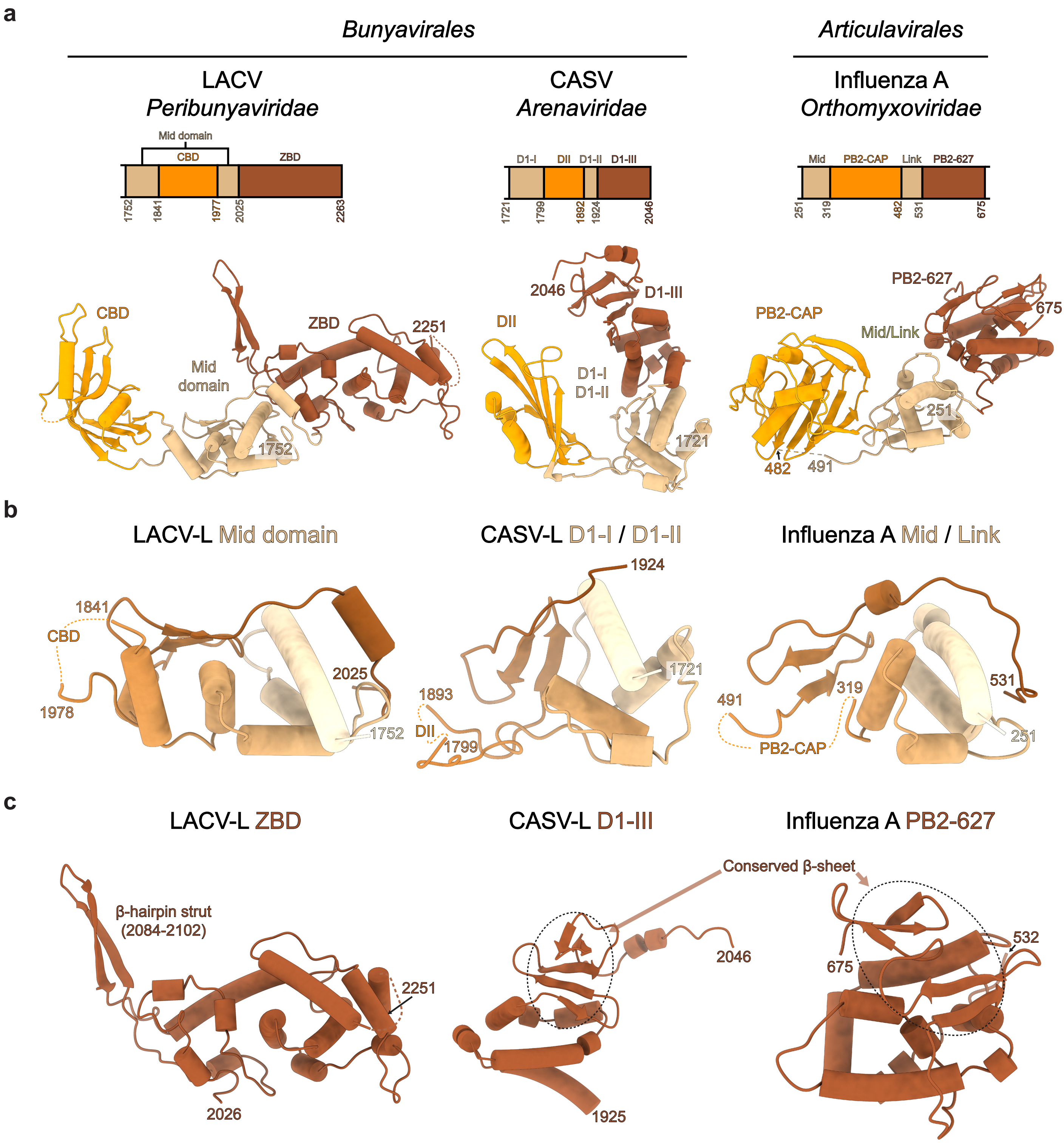

Supplementary figure 7: Comparison of C-terminal domain organization between sNSV polymerases
a, C-terminal domains of LACV-L, CASV-L (PDB: 5MUS) and influenza A virus polymerase (PDB: 4WSB): schematic representation (top) and structure (bottom). Similar domains are colored in the same way. **b**, Superimposed structures of LACV-L mid domain, CASV D1-I/D1-II and influenza A mid/link. Rainbow colors from beige to brown is used to color from the N-terminal to the C-terminal. **c**, Structure of LACV-L ZBD, CASV D1-III and influenza A PB2-627. CASV D1-III and influenza A PB2-627 domains are superimposed. Their conserved β-sheet is surrounded by a dotted line.

Supplementary Figure 8

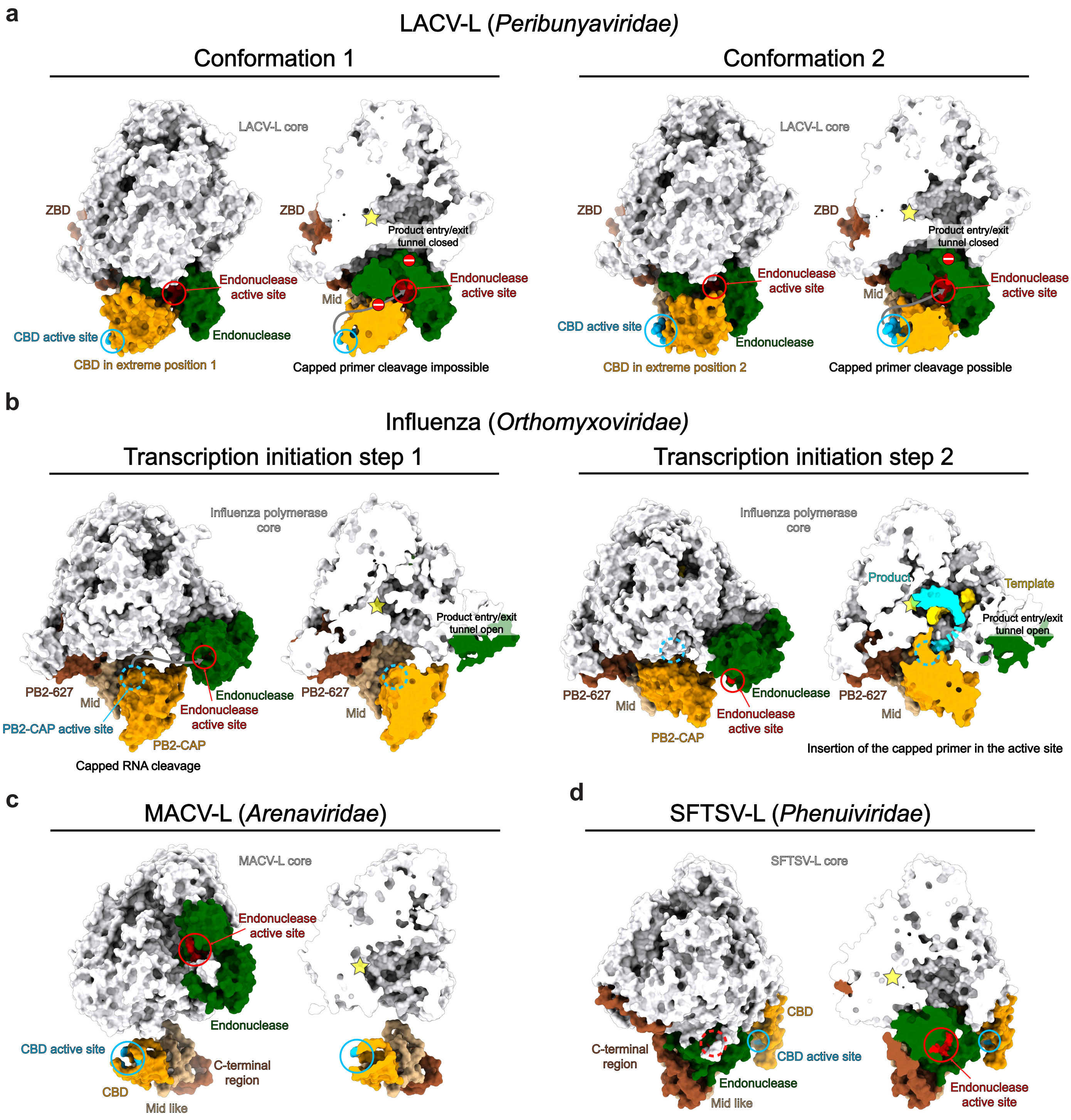

Supplementary figure 8: Position of the endonuclease and C-terminal regions of LACV-L, influenza virus polymerase, Machupo polymerase (MACV-L) and Sever fever with thrombocytopenia syndrome virus polymerase (SFTSV-L)

All polymerase structures are aligned according to the polymerase core and are displayed as molecular surface. For each polymerase and/or conformation, both full and cutted views are displayed. **a**, LACV-L in conformation 1 and 2. LACV-L core is shown in white, the endonuclease in green with its active site in red. LACV-L CBD is colored in orange with its active site in blue. Both mid domain and ZBD are colored respectively in beige and brown. Polymerase core active site location is shown as a yellow star. Grey arrow mimics the path of the bound capped primer through the endonuclease active site. Its access is closed in CBD extreme position 1 (stop sign) and opened in CBD extreme position 2. The product entry/exit tunnel is closed in both conformation (stop sign). **b**, Influenza polymerase in transcription initiation step 1 (PDB: 4WSB) and 2 (PDB: 6QCT). Same colors as in **a** are used for similar and/or related domains. PB2-CAP active site is not visible but represented as a dotted blue circle. Grey arrow mimics the capped RNA cleavage in step 1. In step 2, product and template are respectively colored in cyan and yellow. Product entry/exit tunnel stay open in both conformations. **c**, MACV-L monomer bound to vRNA was extracted from the polymerase dimer (PDB: 6KLH). **d**, SFTSV-L (PDB: 6L42). The endonuclease active site (not visible on the full view) is represented as a dotted red circle.

Supplementary Figure 9

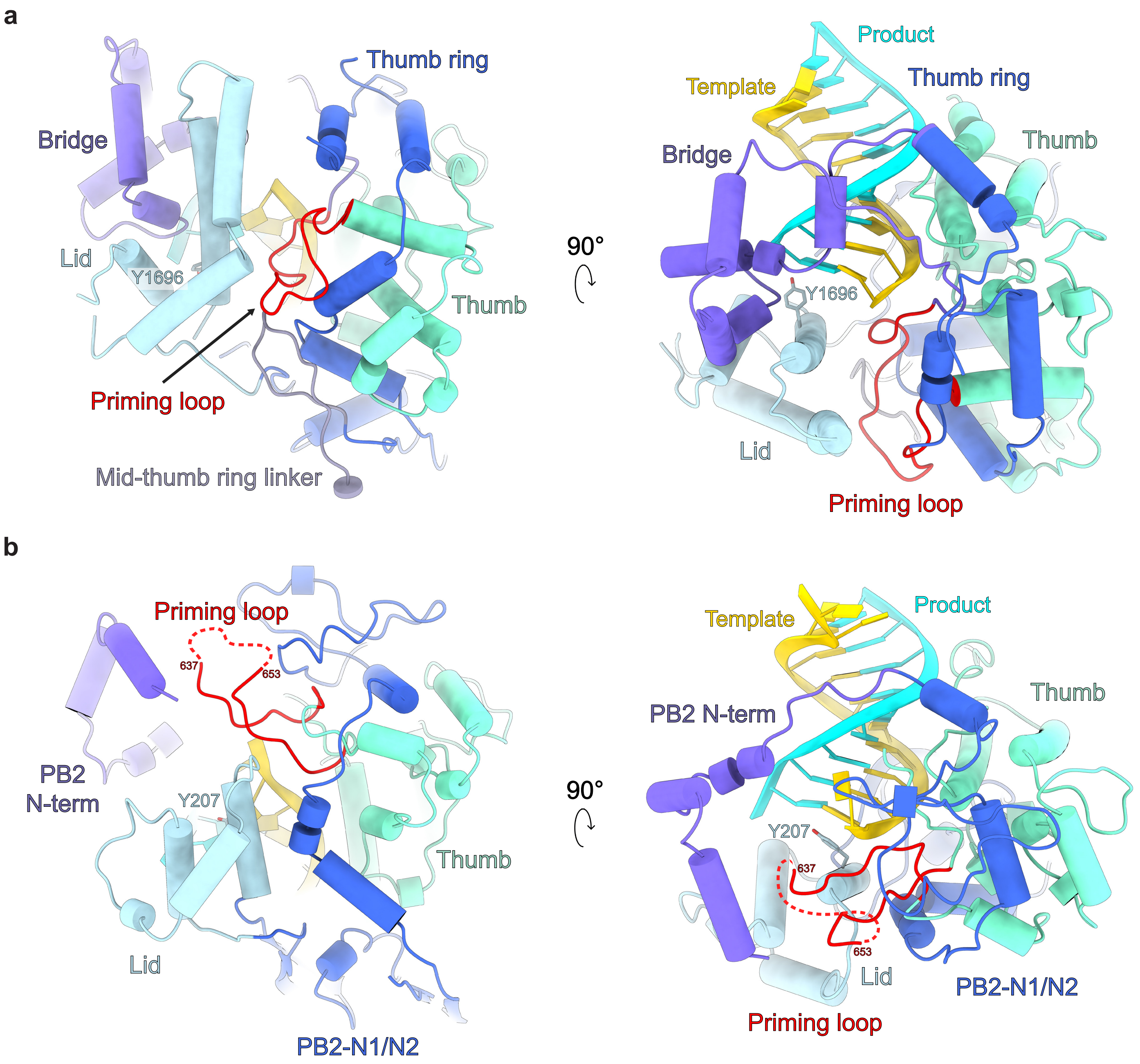

Supplementary figure 9: Comparison between LACV-L and influenza polymerase priming loops at elongation

a, LACV-L thumb, thumb ring, bridge and lid domains are displayed in turquoise, blue, purple and light blue respectively. The priming loop is shown in red. The template RNA is shown in yellow and the product in cyan. dsRNA top view (left) and side view (right) are shown. **b**, Equivalent elements are shown using the same color code in influenza virus polymerase (PDB: 6QCT). The priming loop extremity is disordered and is shown as a dotted line.
