# Supplementary material for: Pre-initiation and elongation structures of full-length La Crosse virus polymerase reveal functionally important conformational changes
Source: Nat Commun. 2020 Jul 17;11:3590. doi: 10.1038/s41467-020-17349-4 (PMC7368059; doi:10.1038/s41467-020-17349-4)
Supplement: Supplementary file 3 — Description of Additional Supplementary Information [file 41467_2020_17349_MOESM3_ESM.pdf]

Description of Additional Supplementary Files

File Name: Supplementary Data 1

Description: **Multiple alignment of Peribunyaviridae L proteins**. Multiple alignment of six Peribunyaviridae L proteins: LACV, Bunyamwera virus (BUNYW), Schmallenberg (SBVBH), Macaua virus (MCAV), Wolkberg virus (WBV) and Oya virus. LACV-L secondary structures are shown and numbered. Domain positions and motifs are indicated. Endonuclease and CBD active site residues are labelled with green and gold stars respectively. Residues that coordinate the zinc are shown with a brown star. Residues that interact with the template/product RNA are labelled with a circle colored based on their domain localization.

File Name: Supplementary Movie 1

Description: **3D variability from cryoSPARC cap-binding domain and zinc-binding domain flexibility.** LACV-L FL sequential cryo-EM reconstructions generated with cryoSPARC 3D variability. LACV-L cryo-EM reconstructions are displayed in the same orientation as in Fig.1b and using the same color code as in Fig.1. Movement of the CBD between the extreme position 1 and the extreme position 2 is visualized. Movement of the ZBD is also depicted.

File Name: Supplementary Movie 2

Description: **3D variability from cryoSPARC, pre-initiation and elongation-mimicking states.** LACV-L FL sequential cryo-EM reconstructions generated with cryoSPARC 3D variability. A surface view and a cut-away view, rotated 90° compared to the surface view, are shown. Domains are colored as in Fig.1. Domains that undergo movements are indicated. The 10-base pair RNA that is either present or absent depending on the reconstruction considered is shown in yellow (template) and blue (product) in the active site cavity. The active site and the four entry/exit tunnels are indicated. The domain movements are depicted.
