# Supplementary material for: Pre-initiation and elongation structures of full-length La Crosse virus polymerase reveal functionally important conformational changes
Source: Nat Commun. 2020 Jul 17;11:3590. doi: 10.1038/s41467-020-17349-4 (PMC7368059; doi:10.1038/s41467-020-17349-4)
Supplement: Supplementary file 4 — Supplementary Data 1 [file 41467_2020_17349_MOESM4_ESM.pdf]

Supplementary Data (1/4)

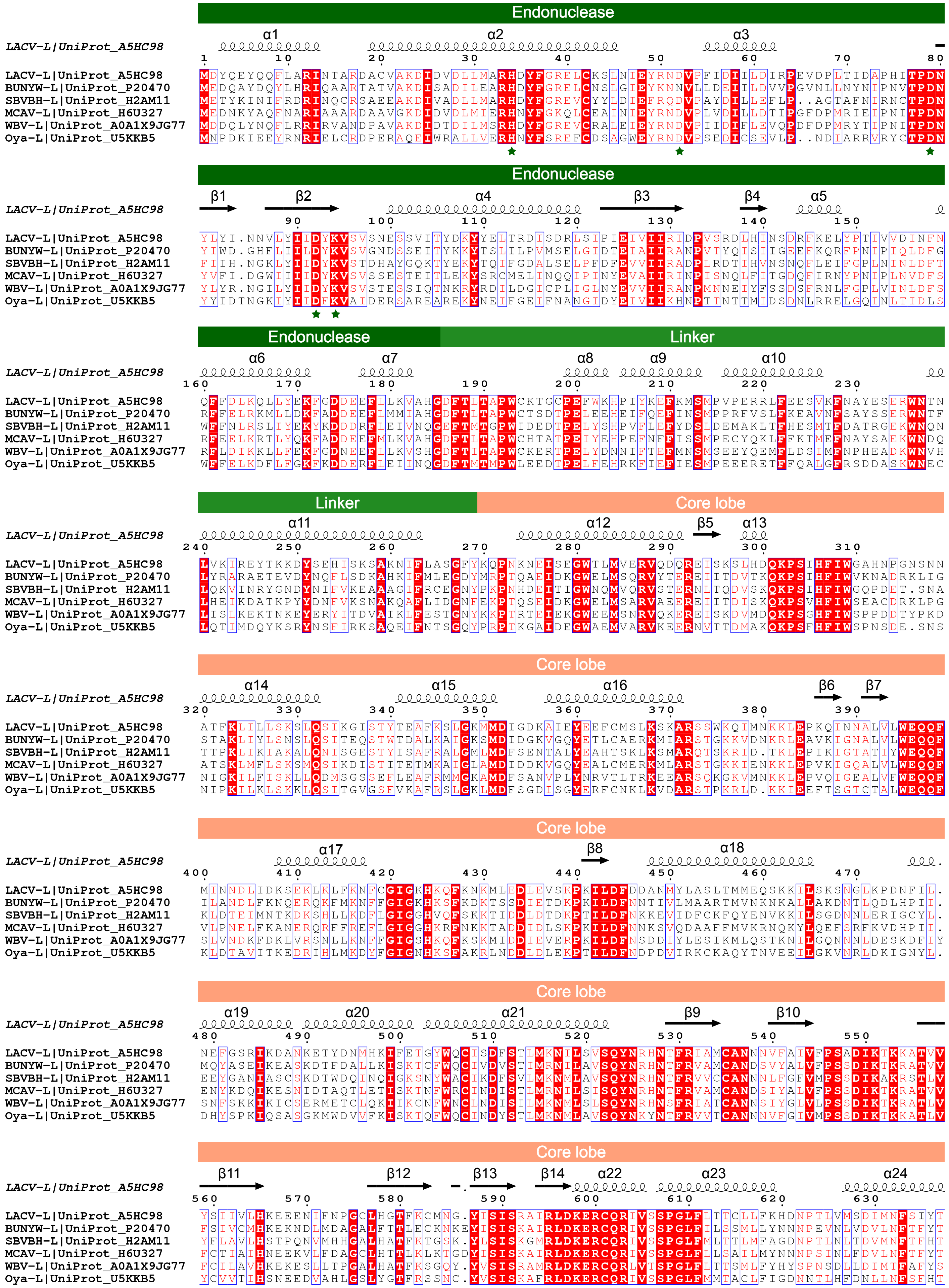

Supplementary Data (2/4)

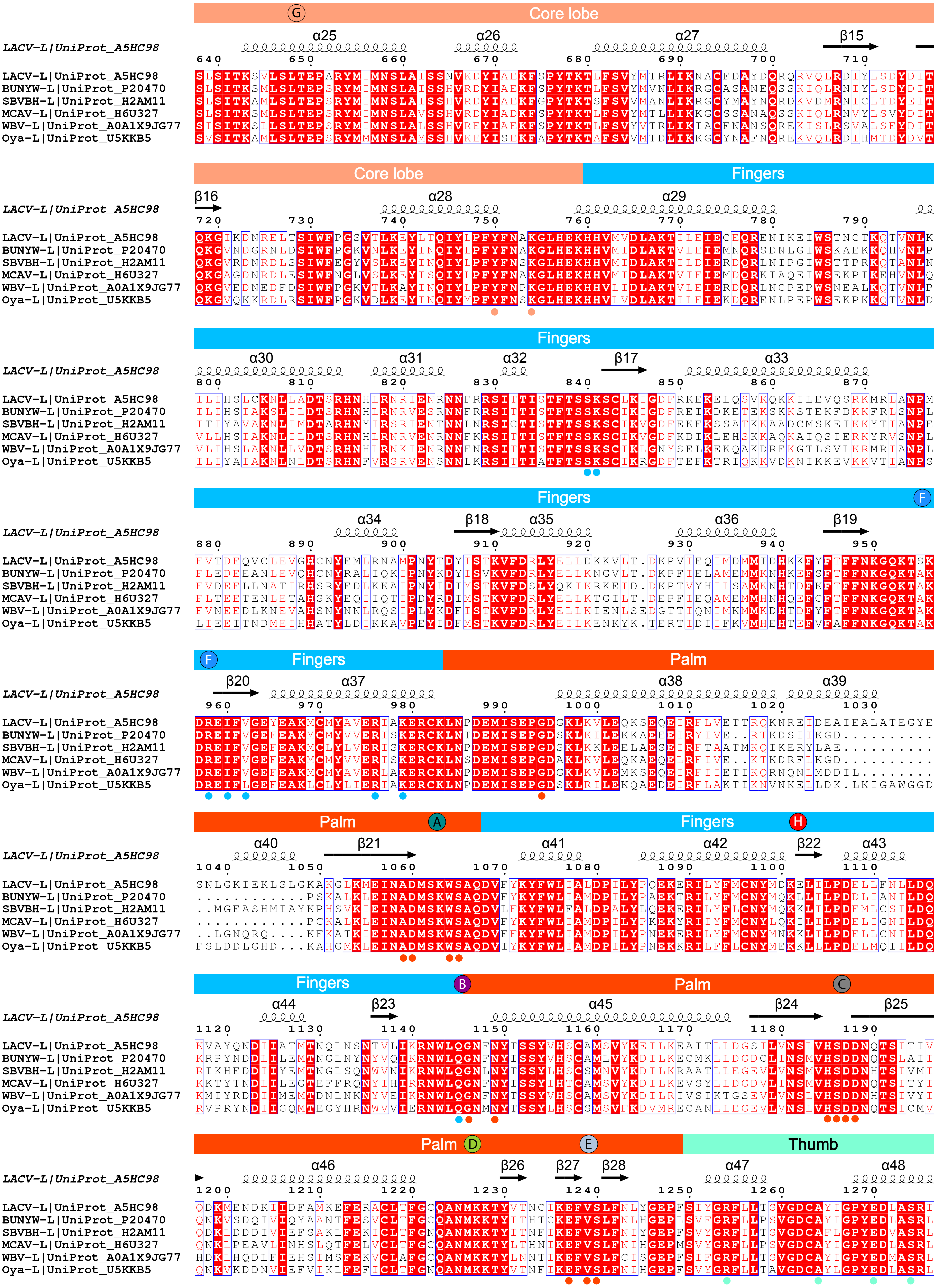

Supplementary Data (3/4)

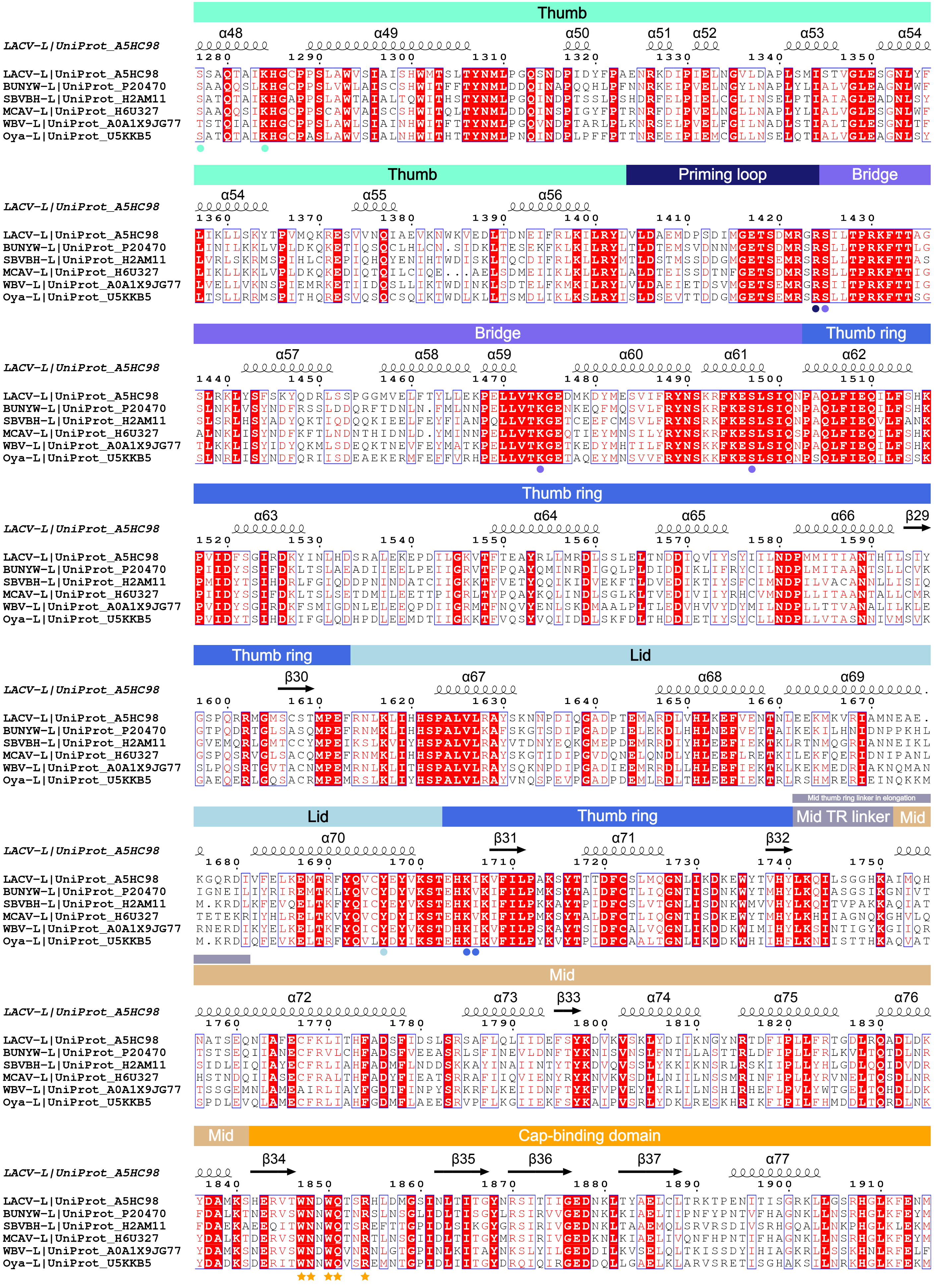

Supplementary Data (4/4)

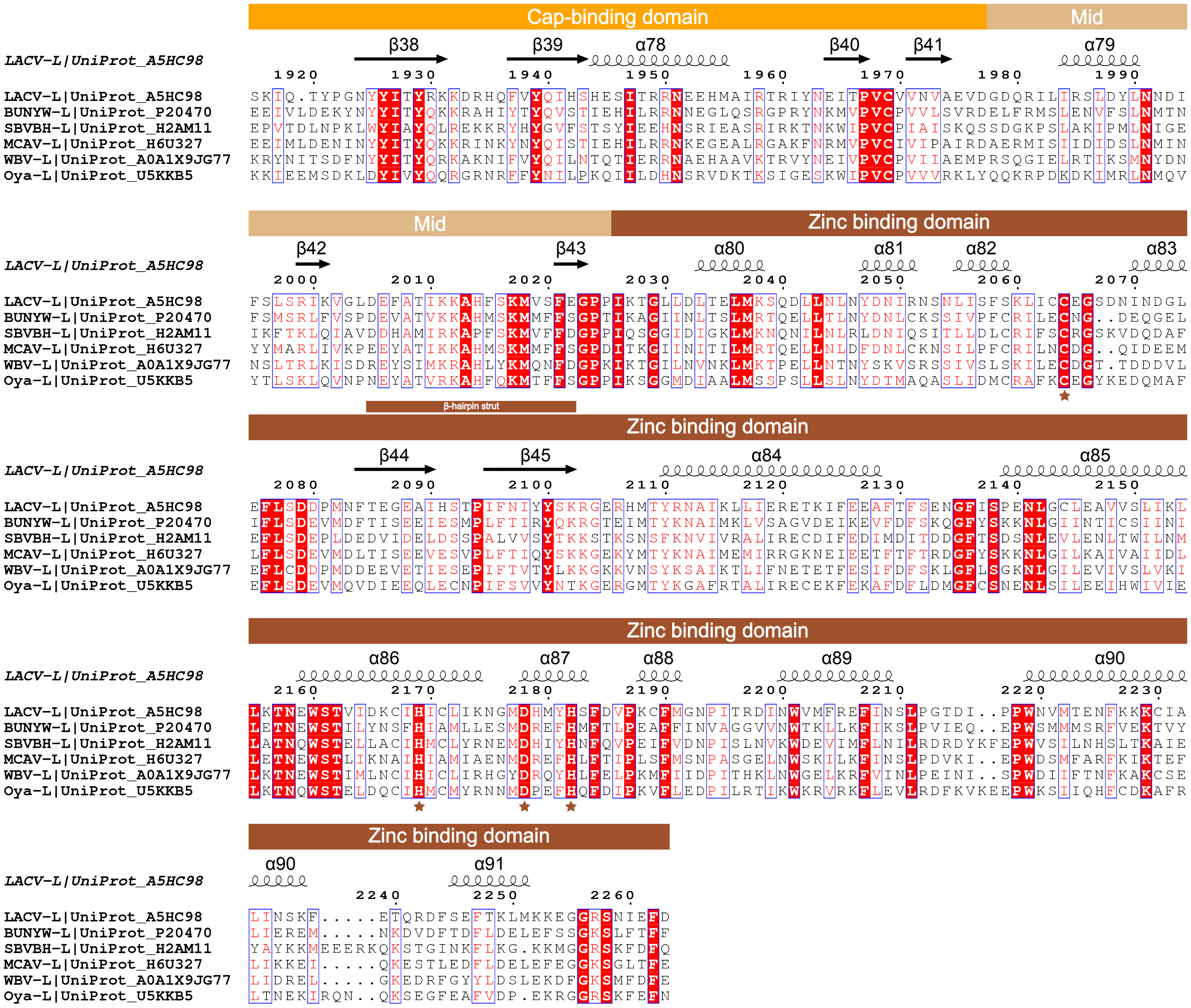

Supplementary data: Multiple alignment of *Peribunyaviridae* L proteins

Multiple alignment of six *Peribunyaviridae* L proteins: LACV, Bunyamwera virus (BUNYW), Schmallenberg (SBVBH), Macaua virus (MCAV), Wolkberg virus (WBV) and Oya virus. LACV-L secondary structures are shown and numbered. Domain positions and motifs are indicated. Endonuclease and CBD active site residues are labelled with green and gold stars respectively. Residues that coordinate the zinc are shown with a brown star. Residues that interact with the template/product RNA are labelled with a circle colored based on their domain localization.
